# 347. Development of an Electronic Medical Record-Based Outpatient Parenteral Antibiotic Therapy (OPAT) Tracking Dashboard

**DOI:** 10.1093/ofid/ofad500.418

**Published:** 2023-11-27

**Authors:** Liddy Bileck, Morgan O’Donnell, Katy Phillips, Courtney Bolton, Ryan Babcock, Elizabeth Herrle, Oliver Fremont, Stephen Rawlings

**Affiliations:** Maine Medical Center, Portland, Maine; Maine Medical Center, Portland, Maine; Maine Medical Center, Portland, Maine; Maine Medical Center, Portland, Maine; Maine Medical Center, Portland, Maine; Maine Medical Center, Portland, Maine; Maine Medical Center, Portland, Maine; Maine Medical Center, Portland, Maine

## Abstract

**Background:**

Patients receiving intravenous (IV) antibiotics in the outpatient setting require intensive laboratory monitoring and frequent clinical intervention. Infectious Diseases (ID) clinics often provide this care due to the need for dedicated staff for lab surveillance and follow-up scheduling. Before April 2023, our OPAT program required a minimum of 3 (non-physician) clinic staff who were tracking patients using a patchwork of paper lists, Electronic Health Record (EHR) “sticky-notes,” and telephone encounter notes.

**Methods:**

From September 2022 to April 2023, infectious disease stakeholders at MaineHealth were engaged in a specialty-specific EHR optimization program and were asked to generate a wish list of improvements. The development of a reliable, user-friendly OPAT tracking tool was identified as a key priority. Dedicated IT analysts searched publicly available databases of tools for our EHR (Epic, Verona, WI) and reviewed existing, local reporting tools to identify solutions. Through an iterative process with ID stakeholders, IT analysts, and informatics specialists, a reporting tool was developed and implemented for all ID specialty offices.

**Results:**

Stakeholders noted three key priorities for OPAT tracking - 1) identifying recently discharged patients on OPAT 2) flagging patients due for interventions (e.g. labs, IV catheter removal, follow-up appointments), and 3) easy viewing of individual patient data to identify unique circumstances and home agency information. A locally-built innovative Reporting Workbench report for tracking pediatric kidney transplant patients served as a model for our build, as no identified tools in the EHR database matched our stakeholder needs.

OPAT Dashboard

**Figure:**
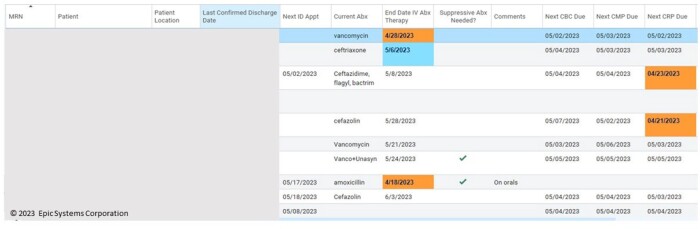

A view of the OPAT dashboard highlighting the ability to track patient follow-up, outstanding labs, antibiotic courses, and end-of-therapy plans. The dashboard contains many areas for customization and because it is built as a report within the EMR, it can be customized to particular work-flows (e.g. sorting by need for scheduling vs. sort by recent discharge or labs due, etc.).

OPAT Enrollment Widget

**Figure:**
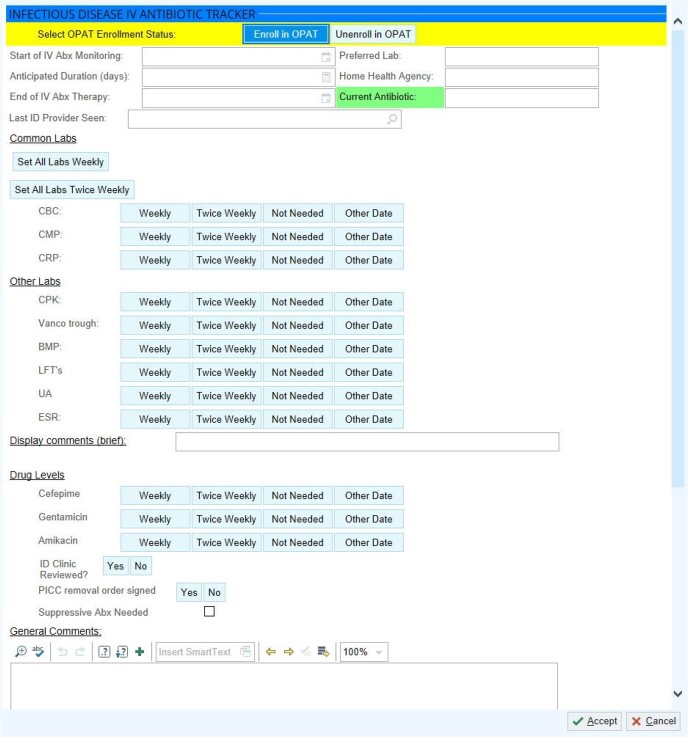

Based on feedback from stakeholders, the enrollment form for this OPAT tracking dashboard allows significant flexibility for lab collection reminders, comments that display in the dashboard, and other functionality. Several fields also do not display on the dashboard for simplicity, but are viewable to users when individual patients are selected in the dashboard.

**Conclusion:**

In the early stages of using this tool, clinic staff have reported increased satisfaction with the new OPAT tracker. The development of this tool highlights the benefit of frequent, collaborative interaction between clinical IT teams, physician informaticists, and subject-matter experts who use the system. The success of this OPAT tracking tool has prompted us to investigate rolling out a similar tool for additional programs including HIV/PrEP care and Hepatitis C treatment.

**Disclosures:**

**All Authors**: No reported disclosures

